# Former Very Preterm Infants Show an Unfavorable Cardiovascular Risk Profile at a Preschool Age

**DOI:** 10.1371/journal.pone.0168162

**Published:** 2016-12-13

**Authors:** Anna Posod, Irena Odri Komazec, Katrin Kager, Ulrike Pupp Peglow, Elke Griesmaier, Elisabeth Schermer, Philipp Würtinger, Daniela Baumgartner, Ursula Kiechl-Kohlendorfer

**Affiliations:** 1 Department of Pediatrics, Pediatrics II (Neonatology), Medical University of Innsbruck, Innsbruck, Austria; 2 Department of Pediatrics, Pediatrics III (Pediatric Cardiology, Pulmonology, Allergology and Cystic Fibrosis), Medical University of Innsbruck, Innsbruck, Austria; 3 Central Institute for Medical and Chemical Laboratory Diagnosis, Innsbruck University Hospital, Innsbruck, Austria; 4 Department of Pediatrics, Clinical Division for Cardiology, Medical University of Graz, Graz, Austria; Centre Hospitalier Universitaire Vaudois, FRANCE

## Abstract

Cardiovascular disease is the leading cause of death worldwide. Evidence points towards an unfavorable cardiovascular risk profile of former preterm infants in adolescence and adulthood. The aim of this study was to determine whether cardiovascular risk predictors are detectable in former very preterm infants at a preschool age. Five- to seven-year-old children born at <32 weeks’ gestational age were included in the study. Same-aged children born at term served as controls. Basic data of study participants were collected by means of follow-up databases and standardized questionnaires. At study visit, anthropometric data, blood pressure readings and aortic intima-media thickness were assessed. Blood samples were obtained after an overnight fast. In comparison to children born at term, former preterm infants had higher systolic and diastolic blood pressure readings (odds ratio [95% confidence interval] per 1-SD higher blood pressure level 3.2 [2.0–5.0], p<0.001 and 1.6 [1.1–1.2], p = 0.008), fasting glucose levels (OR [95% CI] 5.2 [2.7–10.1], p<0.001), homeostasis model assessment index (OR [95% CI] 1.6 [1.0–2.6], p = 0.036), and cholesterol levels (OR [95% CI] 2.1 [1.3–3.4], p = 0.002). Systolic prehypertension (23.7% vs. 2.2%; OR [95% CI] 13.8 [3.1–60.9], p = 0.001), elevated glucose levels (28.6% vs. 5.9%; OR [95% CI] 6.4 [1.4–28.8], p = 0.016), and hypercholesterolemia (77.4% vs. 52.9%; OR [95% CI] 3.0 [1.3–7.1], p = 0.010) were significantly more prevalent in the preterm group. As former very preterm infants display an unfavorable cardiovascular risk profile already at a preschool age, implementation of routine cardiovascular follow-up programs might be warranted.

## Introduction

Due to advances in perinatal care, survival rates of infants born preterm have continuously improved during the last decades [1]. With an increasing number of former preterm infants now reaching adulthood without major overt morbidity, new health challenges arise [2].

Cardiovascular disease is the leading cause of death worldwide and its foundation is thought to be formed early in life [3,4]. The detrimental effect of small birth size is already well established [4]. In addition, an increasing body of evidence suggests that prematurity per se is associated with an unfavorable cardiovascular risk profile in adolescent and adult life, but respective data for children are sparse [5].

Traditional cardiovascular risk factors include modifiable risk factors such as lifestyle factors, overweight/obesity, high blood pressure, hyperglycemia, and lipid alterations [6]. Additional parameters have been shown to be useful in risk assessment, including aortic intima-media thickness (IMT) as a noninvasive marker of preclinical atherosclerosis especially in high-risk children, and adipocytokines, particularly the antiatherosclerotic adiponectin and the proatherogenic leptin [7,8].

The aim of this study was to assess whether known major cardiovascular risk factors and additional indicators of an increased cardiovascular risk are present in former very preterm infants at a preschool age in comparison to same-aged children born at term.

## Materials and Methods

### Study design and population

This study was carried out at the Department of Pediatrics, Innsbruck University Hospital, Austria, from May 2012 to March 2015. The survey area, Tyrol, is a state in Western Austria with 680 000 inhabitants and approximately 7000 live births per year. We investigated a group of former preterm infants born between 01/01/2007 and 07/31/2009 at <32 weeks’ gestational age, who were invited to a routine preschool visit at our preterm follow-up clinic, and a control group of same-aged children born at term who were recruited through regional kindergartens or while undergoing routine preoperative screening for common surgical procedures (adenotomy, tonsillotomy) at Innsbruck University Hospital. None of the subjects had congenital malformations or chromosomal abnormalities. Approval of this study by the local ethics committee, IRB Medical University of Innsbruck (UN 4491), was obtained in advance. Written informed consent was obtained from all legal guardians. All children included in the study orally consented to participation.

### Perinatal characteristics

Basic perinatal data for each child belonging to the preterm study group were drawn from the routine preterm follow-up database at our institution. To account for gender-and gestational age-specific differences, birth weight z-scores were calculated for every subject by means of the Fenton 2013 Growth Calculator for Preterm Infants (available from http://www.peditools.org/fenton2013) [9]. Classification of smoking during pregnancy was based on self-reporting by mothers. Maternal educational status was classified as less than 12 years or 12 years and more.

### Study visit

All examinations were carried out either at Innsbruck University Hospital or at provisional medical posts installed in participating kindergartens between 8 a.m. and 10 a.m. by specifically trained personnel. After a routine clinical examination, weight was measured by means of calibrated medical precision scales and height was determined by a Harpenden stadiometer. Body mass index (BMI) was calculated as weight in kilograms divided by height in meters squared. In order to account for gender- and age-specific differences, BMI z-scores were calculated for each study participant by means of a reference data set[10].

Blood pressure was measured thrice on the right upper arm with the appropriate cuff size after a five-minute resting period in a seated position using an automated oscillometric device.

Intima-media thickness (IMT) was determined ultrasonographically in the dorsal arterial wall of the most caudal 15 mm of the abdominal aorta by means of a high frequency linear array transducer (12L-RS, GE Healthcare) as described previously [7].

Blood samples were collected after a minimum overnight fasting period of eight hours. Fasting glucose, fasting insulin, total cholesterol, low-density lipoprotein (LDL) cholesterol, high-density lipoprotein (HDL) cholesterol, triglyceride, adiponectin and leptin levels were determined at the Central Institute for Medical and Chemical Laboratory Diagnosis (Innsbruck University Hospital). Homeostasis model assessment (HOMA) index was calculated for each subject as fasting insulin [mU/l] times fasting glucose [mg/dl], divided by 405.

All children and their parents were asked to fill out a questionnaire regarding family history of cardiovascular disease (CVD) and childhood nutrition habits. A positive family history of CVD was defined as a diagnosis of coronary heart disease, angina, heart attack, congenital heart disease or stroke in first-degree male relative of child and/or parent under the age of 55 or first-degree female relative of child and/or parent under the age of 65 [11]. Information on childhood nutrition habits was gathered by means of an established standardized food frequency questionnaire (“What do you eat?”, kindly provided by the Robert Koch Institute, Berlin, Germany) [12]. Childhood nutrition was subsequently categorized as unfavorable, neutral or favorable for further analyses.

In children born at term not previously registered at our hospital, basic perinatal data were collected at study visit. In the preterm group, data not available from our routine preterm follow-up data base were also filled in during the study visit. Remaining missing data were classified as “unknown”.

### Statistical methods

All statistical analyses were performed by means of SPSS for Windows, version 22 (SPSS Inc.).

In order to ensure representativeness of study samples, both term and preterm study cohorts were compared with reference populations (term: SIDS database Tyrol, birth years 2007–2009; preterm: Innsbruck routine preterm follow-up database, birth years 2007–2009) in regard to gender distribution, gestational age, birth weight and maternal educational status by Pearson’s Chi-Square and Mann-Whitney U test.

Differences in perinatal characteristics as well as characteristics at study visit between term and preterm study groups were determined by means of Pearson’s Chi-Square, Mann-Whitney U or Student’s T-test, depending on type and distribution of the variable analyzed.

Logistic regression analysis was used to determine differences between preterm and term subjects in blood pressure and aortic IMT measurements as well as laboratory markers. To account for skewed distributions or heteroscedasticity of the dependent variable, fasting glucose and insulin levels, HOMA index as well as triglyceride, adiponectin and leptin levels were logarithmically transformed prior to analyses. With regard to covariates and potential confounders, a step-wise approach was used. Model A was adjusted for age at examination and gender, birth weight z-score, smoking during pregnancy, maternal educational status and positive family history of CVD. Model B added current BMI z-scores and childhood nutrition profiles. Odds ratios (OR) and respective confidence intervals (CI) were calculated as OR per standard deviation (SD) increase for all continuous variables.

Prevalence of prehypertension (systolic and/or diastolic blood pressure ≥90th percentile), raised fasting glucose (≥75th percentile) and HOMA index (≥75th percentile), elevated total cholesterol (≥150 mg/dl or 3.9 mmol/l) and LDL cholesterol levels (≥100 mg/dl or 2.6 mmol/l) as well as low HDL cholesterol levels (<40 mg/dl or 1.0 mmol/l) was calculated for preterm and term study groups [13,14,15]. OR and respective CI were determined by logistic regression analysis with respective adjustments in models A and B (see above).

## Results

### Study population and demographics

**Fig 1:**
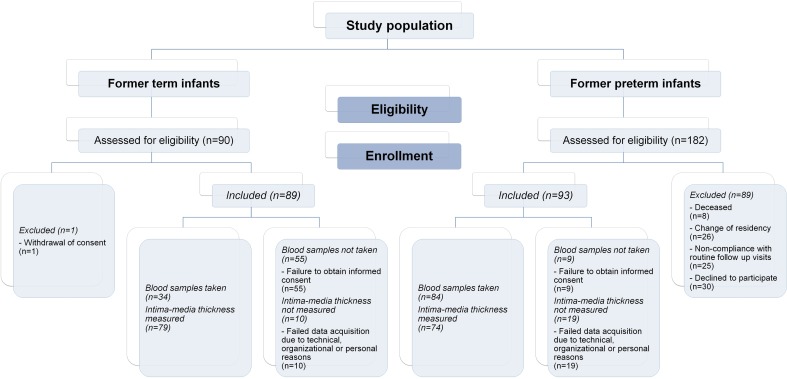
Flow diagram. Overview of children assessed for eligibility and enrolled in the study as well as data availability with regard to laboratory markers and intima-media thickness in term and preterm study groups. Basic perinatal characteristics, anthropometric data and blood pressure measurements were available for all subjects included in the study.

A total number of 90 former term and 182 former preterm infants were assessed for eligibility. 89 former term and 93 former preterm infants were included in the study (Fig 1).

Children born at term enrolled in the study were similar to the general Tyrolean population in birth weight and gender distribution. Maternal educational status was higher in the study cohort. Former preterm infants enrolled in the study did not significantly differ from a preterm reference population in gestational age, birth weight, gender distribution or maternal educational status (data available on request).

### Population characteristics

Perinatal characteristics as well as characteristics at study visit of all children enrolled are given in Table 1. At study visit, children born preterm had a significantly lower body mass index and BMI z-scores than term controls. A smaller percentage of favorable childhood nutrition profiles was reported in this group.

**Table 1:** Perinatal characteristics and characteristics at study visit in former term and preterm infants.

Characteristic	Term group (n = 89)	Preterm group (n = 93)
Gender, male/female [%]	47.2/52.8	52.7/47.3
***Perinatal characteristics***		
Gestational age, mean (SD) [weeks]	39.7 (1.2)	29.3 (2.2)^a^
Birth weight, mean (SD) [grams]	3328 (400)	1209 (397)^a^
Birth weight z-score, mean (SD)	-0.226 (0.831)	-0.214 (0.923)
Small for gestational age at birth [n;%]	6;6.7	11;11.8
Smoking during pregnancy, unknown/yes/no [%]	18.0/10.1/71.9	0.0/25.8/74.2^b^
Maternal educational status, unknown/<12 years/≥12 years [%]	15.7/39.3/44.9	1.1/55.9/43.0
Mainly (>50%) breastfed, unknown/yes/no [%]	4.5/65.2/30.3	1.1/79.6/19.4
***Characteristics at study visit***		
Age at examination, mean (SD) [years]	5.7 (0.5)	5.4 (0.3)^a^
Current BMI, median (IQR) [kg/m^2^]	14.9 (1.5)	14.1 (1.3)^a^
Current BMI z-score, mean (SD)	-0.178 (0.837)	-0.923 (1.263)^a^
Positive family history of CVD, unknown/yes/no [%]	13.5/5.6/80.9	2.2/14.0/83.9
Childhood nutrition profile, unknown/neutral/favorable [%]	6.7/36.0/57.3	9.7/50.5/39.8^c^^†^

Abbreviations: BMI, body mass index (calculated as weight in kilograms divided by height in meters squared); CVD, cardiovascular disease (positive family history of CVD defined as diagnosis of coronary heart disease, angina, heart attack, congenital heart disease or stroke in first-degree male relative of child and/or parent under the age of 55 or first-degree female relative of child and/or parent under the age of 65); IQR, interquartile range; SD, standard deviation.

^a^ p<0.001, term versus preterm group.

^b ^p<0.05, term versus preterm group (yes/no).

^c^ p<0.05, term versus preterm group (neutral/favorable).

^†^ Unfavorable childhood nutrition habits were not reported in either study group.

None of the participants were taking medication with a possible influence on the cardiovascular system, glucose homeostasis or lipid metabolism.

With regard to antenatal administration of corticosteroids, 76.3% of preterm infants received a full course prior to birth and 11.8% received an incomplete course. No corticosteroids were administered in 8.6% of cases; in 3.2% administration was not documented. Postnatal corticosteroids were administered in 14.0% of preterm infants for treatment of bronchopulmonary dysplasia. All findings did not significantly differ from a preterm reference population (p = 1.000 for both analyses).

### Blood pressure and aortic intima-media thickness measurements

In comparison to same-aged control subjects born at term, former preterm infants had higher systolic blood pressure readings (Table 2). Results remained similar after adjusting for covariates in all models applied (Table 3). No differences in mean blood pressures were observed (Tables 2 and 3). With regard to diastolic blood pressure, former preterm infants had higher measured values than children born at term (Table 2) and this difference was significant in the fully adjusted model (Table 3). Aortic IMT was similar in both study groups (Tables 2 and 3).

**Table 2:** Blood pressure readings, aortic intima-media thickness and laboratory parameters.

Variable	Term group (n = 34–89)	Preterm group (n = 74–84)
***Blood pressure readings***		
Systolic, mean (SD) [mmHg]	96 (6)	102 (7)^a^
Mean, mean (SD) [mmHg]	72 (8)	70 (7)
Diastolic, mean (SD) [mmHg]	53 (7)	56 (7)^b^
***Aortic IMT***		
IMT, mean (SD) [mm]	0.464 (0.042)	0.461 (0.055)
***Glucose homeostasis***		
Fasting glucose, mean (SD) [mg/dl]/[mmol/l]	69.7 (12.2)/3.9 (0.7)	83.1 (8.6)/4.6 (0.5)^a^
Fasting insulin, mean (SD) [mU/l]	4.8 (4.9)	5.5 (4.8)
HOMA index	0.89 (0.99)	1.17 (1.19)
***Lipid profiles***		
Total cholesterol, mean (SD) [mg/dl]/[mmol/l]	150.4 (26.8)/3.9 (0.7)	169.5 (28.7)/4.4 (0.7)^b^
LDL cholesterol, mean (SD) [mg/dl]/[mmol/l]	90.4 (20.3)/2.3 (0.5)	101.3 (22.5)/2.6 (0.6)^c^
HDL cholesterol, mean (SD) [mg/dl]/[mmol/l]	52.6 (12.1)/1.4 (0.3)	63.4 (13.9)/1.6 (0.4)^a^
Triglycerides, mean (SD) [mg/dl]/[mmol/l]	61.3 (29.2)/0.7 (0.3)	59.5 (30.1)/0.7 (0.3)
***Adipocytokines***		
Adiponectin, mean (SD) [μg/l]	13068.0 (3242.6)	12754.8 (4876.4)
Leptin, mean (SD) [μg/l]	0.57 (0.86)	1.26 (2.01)

Abbreviations: HDL, high-density lipoprotein; HOMA, homeostasis model assessment (index calculated as fasting insulin [mU/l] times fasting glucose [mg/dl], divided by 405); IMT, intima-media thickness; LDL, low-density lipoprotein.

^a ^p<0.001, term versus preterm group.

^b^ p<0.01, term versus preterm group.

^c^ p<0.05, term versus preterm group.

**Table 3:** Logistic regression analysis of cardiovascular risk factors in children born preterm in comparison to same-aged controls born at term.

Outcome variable and analysis model^b^	β coefficient	p value	OR (95% CI)^a^
***Basic cardiovascular parameters***			
*Systolic blood pressure*^*c*^			
Unadjusted	1.155	***<0*.*001***	3.2 (2.0,5.0)
Model A	1.092	***<0*.*001***	3.0 (1.7,5.1)
Model B	1.393	***<0*.*001***	4.0 (2.1,7.5)
*Mean blood pressure*^*c*^			
Unadjusted	-0.187	0.236	0.8 (0.6,1.1)
Model A	-0.171	0.366	0.8 (0.6,1.2)
Model B	-0.036	0.860	1.0 (0.6,1.4)
*Diastolic blood pressure*^*c*^			
Unadjusted	0.441	***0*.*008***	1.6 (1.1,2.2)
Model A	0.359	0.071	1.4 (1.0,2.1)
Model B	0.451	***0*.*033***	1.6 (1.0,2.4)
*Aortic intima-media thickness*^*c*^			
Unadjusted	-0.067	0.681	0.9 (0.7,1.3)
Model A	-0.129	0.516	0.9 (0.6,1.3)
Model B	-0.148	0.526	0.9 (0.5,1.4)
***Glucose homeostasis***			
*Fasting glucose*^*c*^^,^^*d*^			
Unadjusted	1.644	***<0*.*001***	5.2 (2.7,10.1)
Model A	1.643	***<0*.*001***	5.2 (2.5,10.5)
Model B	1.700	***<0*.*001***	5.5 (2.6,11.6)
*Fasting insulin*^*c*^^,*d*^			
Unadjusted	0.304	0.175	1.4 (0.9,2.1)
Model A	0.326	0.211	1.4 (0.8,2.3)
Model B	0.473	0.110	1.6 (0.9,2.9)
*HOMA index*^*c*^^,^^*d*^			
Unadjusted	0.496	***0*.*036***	1.6 (1.0,2.6)
Model A	0.529	0.053	1.7 (1.0,2.9)
Model B	0.700	***0*.*026***	2.0 (1.1,3.7)
***Lipid profiles***			
*Total cholesterol*^*c*^			
Unadjusted	0.744	***0*.*002***	2.1 (1.3,3.4)
Model A	0.767	***0*.*003***	2.2 (1.3,3.6)
Model B	0.909	***0*.*001***	2.5 (1.4,4.3)
*LDL cholesterol*^*c*^			
Unadjusted	0.539	***0*.*019***	1.7 (1.1,2.7)
Model A	0.500	***0*.*039***	1.6 (1.0,2.7)
Model B	0.602	***0*.*017***	1.8 (1.1,3.0)
*HDL cholesterol*^*c*^			
Unadjusted	0.898	***<0*.*001***	2.5 (1.5,4.0)
Model A	1.046	***<0*.*001***	2.8 (1.6,5.1)
Model B	1.193	***<0*.*001***	3.3 (1.7,6.2)
*Triglycerides*^*c*^^,^^*d*^			
Unadjusted	-0.057	0.780	0.9 (0.6,1.4)
Model A	-0.034	0.876	1.0 (0.6,1.5)
Model B	-0.015	0.949	1.0 (0.6,1.5)
***Adipocytokines***			
*Adiponectin*^*c*^^,^^*d*^			
Unadjusted	-0.152	0.512	0.9 (0.5,1.4)
Model A	-0.152	0.563	0.9 (0.5,1.4)
Model B	-0.072	0.806	0.9 (0.5,1.6)
*Leptin*^*c*^^,^^*d*^			
Unadjusted	0.319	0.205	1.4 (0.8,2.3)
Model A	0.430	0.158	1.5 (0.8,2.8)
Model B	1.227	***0*.*007***	3.4 (1.4,8.3)

Abbreviations: BMI, body mass index (calculated as weight in kilograms divided by height in meters squared); CI, confidence interval; CVD, cardiovascular disease (positive family history of CVD defined as diagnosis of coronary heart disease, angina, heart attack, congenital heart disease or stroke in first-degree male relative of child and/or parent under the age of 55 or first-degree female relative of child and/or parent under the age of 65); HDL, high-density lipoprotein; HOMA, homeostasis model assessment (index calculated as fasting insulin [mU/l] times fasting glucose [mg/dl], divided by 405); LDL, low-density lipoprotein; OR, odds ratio.

^a^ Odds ratios were calculated for former preterm infants in relation to same-aged children born at term.

^b^ Model A was adjusted for age at examination and gender, birth weight z-score, smoking during pregnancy, maternal educational status and positive family history of CVD. Model B was adjusted for all parameters of model A plus current BMI z-score, and childhood nutrition profile.

^c^ Odds ratios are reported as odds ratio per SD increase.

^d^ Input data were logarithmically transformed for analyses.

### Glucose homeostasis

Fasting glucose levels were significantly higher in former preterm infants in comparison to children born at term (Table 2), with consistent results after adjusting for covariates in all statistical models used (Table 3). Fasting insulin levels were higher in the preterm group, but this finding did not reach statistical significance in any of the models applied (Tables 2 and 3). HOMA index was significantly higher in the preterm study group (Table 2). Statistical significance was also reached in the fully adjusted model (Table 3).

### Lipid profiles

In comparison to children born at term, former preterm infants had significantly higher total cholesterol and LDL cholesterol, but also HDL cholesterol levels (Table 2). Results were similar throughout all statistical models (Table 3). Triglyceride levels were similar in both groups (Tables 2 and 3).

### Adipocytokine levels

No differences between groups were found in adiponectin levels (Tables 2 and 3). With regard to leptin, higher levels were observed in the preterm study group in comparison to term controls. This finding reached statistical significance after adjusting for current BMI z-scores, and childhood nutrition profiles (Table 3).

### Prevalence of cardiovascular risk factors at preschool age

In order to obtain a better understanding of the clinical relevance of our findings, we also determined the prevalence of manifest abnormalities in blood pressure, glucose and lipid metabolism. In accordance with above-mentioned results, the prevalence of systolic prehypertension was significantly higher in former preterm infants in comparison to term-born controls (23.7% vs. 2.2%; OR [95% CI] 13.8 [3.1–60.9], p = 0.001). Prevalence of diastolic prehypertension was also higher in the preterm group, but this finding did not reach statistical significance. Results were similar after adjustment for covariates in all statistical models.

Raised fasting glucose levels were more frequently observed in the preterm group (28.6% vs. 5.9%; OR [95% CI] 6.4 [1.4–28.8], p = 0.016; similar results in models A and -B). Also, raised HOMA index was more common in former preterm infants (49.4% vs. 27.6%; OR [95% CI] 2.6 [1.0–6.4], p = 0.046; statistical significance also reached in model B).

With regard to hypercholesterolemia, total cholesterol levels above the normal range were significantly more prevalent in the preterm study group (77.4% vs. 52.9%; OR [95% CI] 3.0 [1.3–7.1], p = 0.010); similar results in models A and B). Comparable results were found for elevated LDL cholesterol levels (54.8% vs. 26.5%; OR [95% CI] 3.4 [1.4–8.1]; p = 0.007; similar results in models A and -B). The prevalence of low HDL cholesterol levels was similar between groups (3.7% vs. 9.7%; OR [95% CI] 0.4 [0.07–2.0], p = 0.255; similar results in models A and B).

Due to the small number of small for gestational age newborns in both study groups, a subgroup analysis for these subjects was not undertaken. A separate analysis excluding small for gestational age subjects was conducted and yielded similar results (comparable effect sizes) in all regards.

Blood pressure readings, aortic IMT measurements, fasting glucose and insulin levels, HOMA index, total, LDL and HDL cholesterol levels, triglyceride, adiponectin and leptin levels were not influenced by use of antenatal corticosteroids and did not significantly differ between former preterm infants who did or did not receive corticosteroids postnatally (S1 and S2 Tables).

In preterm-born children heightened risk factor levels were independent of gestational age. For example, mean systolic blood pressure readings were 101, 101, 106, 100, and 101 mmHg in gestational age groups <26 weeks, 26–28 weeks, 29 weeks, 30 weeks, and 31–32 weeks. Mean fasting glucose levels were 81 mg/dl (4.5 mmol/l), 82 mg/dl (4.6 mmol/l), 83 mg/dl (4.6 mmol/l), 87 mg/dl (4.8 mmol/l), and 83 mg/dl (4.6 mmol/l); total cholesterol levels were 158 mg/dl (4.1 mmol/l), 164 mg/dl (4.2 mmol/l), 175 mg/dl (4.5 mmol/l), 176 mg/dl (4.6 mmol/l), and 171 mg/dl (4.4 mmol/l).

## Discussion

As an increasing number of former preterm infants nowadays are reaching adolescence and adulthood without major overt morbidity, research focus has recently shifted towards longer-term implications of premature birth. A growing body of evidence points towards a negative effect of prematurity on future cardiovascular health. As early detection of markers of an increased cardiovascular risk is crucial in order to implement effective preventive strategies, the aim of the current study was to evaluate whether former preterm infants show alterations in known major modifiable and additional cardiovascular risk factors already at a preschool age in comparison to same-aged children born at term.

The preschool period was chosen for this study, because at five to seven years of age lifestyle factors such as tobacco use and alcohol consumption are absent. In addition, due to fairly standardized exercise programs in Austrian kindergartens, less variability in the range of physical activity is to be expected.

At study visit, children born preterm had significantly lower current BMI z-scores than children born at term, even though birth weight z-scores were similar in both groups. This is in accordance with growth trajectories published by Saigal et al. who reported decreasing weight z-scores between birth and one year of age, and BMI z-scores below zero at three and eight years of age in extremely low birth weight preterm infants [16]. Of interest, Saigal at el. described a subsequent catch-up in mean BMI z-scores with crossing of percentiles in both sexes. This finding is corroborated by other studies indicating increased obesity rates in adults born preterm [17,18]. Whether the preterm infants examined in our study develop obesity later in life as suggested by literature has to be evaluated in follow-up studies.

With regard to blood pressure, former preterm infants had significantly higher systolic and diastolic blood pressures than same-aged children born at term. This is in accordance with meta-analyses reporting higher blood pressure measurements in former preterm adolescents and adults [19,20]. To the contrary, the EPICure study reported lower systolic and diastolic blood pressure readings in preterm infants in comparison to matched term-born controls at six years of age, but differences became non-significant after adjustment for covariates [21,22]. As systolic hypertension is known to be a strong predictor of cardiovascular events, prodromal elevations of systolic blood pressure might be of particular relevance as a focus of future CVD prevention programs in former preterm infants [6]. Interestingly, aortic IMT did not differ between former preterm infants and term-born controls. This is in contradiction to the findings of Shimizu et al., who reported thickening of the aortic IMT in former preterm infants in comparison term-born controls at a preschool age [23]. However, in their study, the ventral wall of the abdominal aorta was used for measurements instead of the dorsal wall, and also, a lower-frequency transducer was applied, thus limiting comparability [7,23]. Also, due to the rather small sample size in that trial, generalizability of data is reduced. It is noteworthy that overall aortic IMT measurements were higher in Shimizu et al.’s sample than in our study population. This might be ascribable to ethnic differences, as Shimizu et al. studied a Japanese only population. Even though no differences were observed between groups in our study, follow-up at a more advanced age might still be warranted, as longer-term effects of prematurity on the vasculature are not unlikely [24].

As prematurity has been associated with adult-onset type 2 diabetes and children born prematurely have been shown to have decreased insulin sensitivity upon repeated blood sampling, we evaluated whether alterations in glucose metabolism can be detected at a preschool age with single blood sampling [25,26,27]. In comparison to children born at term, former preterm infants had significantly higher fasting glucose levels (83.1 vs. 69.7 mg/dl, p<0.001). Although fasting insulin levels did not significantly differ between groups, HOMA index was significantly higher in preterm-born children, potentially indicating altered insulin sensitivity. This finding is in discordance with a study published by Darendeliler et al., who report no differences in HOMA index between former preterm and term infants at preschool age [28]. This discrepancy might be explained by the fact that Darendeliler et al. included former preterm infants of all gestational ages below 37 weeks, whereas our study focused on the high-risk very preterm population.

To date, data on dyslipidemia in former preterm infants are inconsistent [5]. To the best of our knowledge, we are the first to show elevated levels of total and LDL cholesterol, but also HDL cholesterol in children born very preterm at a preschool age. The exact implications of these “dualistic alterations” in lipid profiles are unknown, as both a negative impact on cardiovascular health of elevated LDL cholesterol, but also a protective effect of high HDL cholesterol levels is possible. In conjunction with an altered glucose homeostasis, however, dyslipidemia poses a substantial threat to cardiovascular health in adult life and its development over time thus merits further investigation in future studies.

With regard to adipocytokines, we did not observe any differences in adiponectin levels. Leptin levels were higher in former preterm infants, even though this finding only reached statistical significance after adjustment for current BMI z-score. As it has been reported that a leptin/adiponectin imbalance plays a particular role in the development of metabolic syndrome and diabetes, both findings are of relevance and may add to the more classical cardiovascular risk factors present in the preterm study cohort [29].

Within the group of preterm-born children, risk factor levels were not related to gestational age. However, statistical power is limited for this type of analysis because of small subgroup sample sizes.

In discordance with a study published by Sipola-Leppänen et al., we did not observe gender-specific differences in cardiovascular risk profiles [30]. This, however, might be explained by the fact that this population-based cohort study examined adolescents, whereas our study focused on the preschool age. It is entirely possible that gender-specific differences also become noticeable in our study population after onset of puberty, as biological sex is considered a major determinant for the development and progression of cardiovascular disease [31]. Longer-term follow-up studies are thus required.

One of the limitations of our study is that of all former preterm infants assessed for eligibility, only 51% could be included in our study, mostly owing to a change in residency or non-compliance with our routine preterm follow-up program. It is thus possible that subjects with impairments associated with preterm birth are underrepresented. However, our cohort did not differ from a preterm reference population in basic perinatal characteristics, thus warranting representativeness of samples.

The main limitation of our study is that due to its design, measurements were only undertaken at a single time point. With regard to exact implications on cardiovascular health in the long run, longitudinal studies with follow-up into school age as well as early and late adulthood are direly needed. As a further limitation, blood samples were available only in a subgroup of term-born children. All other variables, however, were ascertained in the full sample.

Although risk factor levels in absolute terms were within a normal range, quantitative differences between the term and preterm group were partly considerable (e.g. fasting glucose or total cholesterol levels) and clinically meaningful. It should be noted that none of the observations made in our study required immediate treatment or intervention, but alterations prompted timely follow-up in a substantial number of participants. The observed increased odds of manifest blood pressure and cholesterol elevations in former preterm infants in conjunction with the “preclinical” alterations in glucose metabolism might give adequate ground for a paradigm shift in the conceptualization of preterm follow-up programs. Implementation of routine cardiovascular follow-up programs for former very preterm infants might be warranted.

## Supporting Information

S1 Table Effect of antenatal administration of corticosteroids on cardiovascular risk indicators. (DOCX)

S2 Table Effect of postnatal administration of corticosteroids on cardiovascular risk indicators. (DOCX)
