# Supplementary material for: Former Very Preterm Infants Show an Unfavorable Cardiovascular Risk Profile at a Preschool Age
Source: PLoS One. 2016 Dec 13;11(12):e0168162. doi: 10.1371/journal.pone.0168162 (PMC5154574; doi:10.1371/journal.pone.0168162)
Supplement: S1 Table — (DOCX) [file pone.0168162.s001.docx]

**S1 Table. Effect of antenatal administration of corticosteroids on cardiovascular risk indicators**

| **Variable** | | **No use of antenatal corticosteroids**  **(n=5-8)** | **Any use of antenatal corticosteroids**  **(n=64-76)** |
| --- | --- | --- | --- |
| ***Blood pressure readings*** | |  |  |
|  | Systolic, mean (SD) [mmHg] | 101 (3) | 103 (7) |
|  | Mean, mean (SD) [mmHg] | 70 (6) | 71 (7) |
|  | Diastolic, mean (SD) [mmHg] | 58 (6) | 56 (7) |
| ***Aortic IMT*** | |  |  |
|  | IMT, mean (SD) [mm] | 0.437 (0.033) | 0.466 (0.056) |
| ***Glucose homeostasis*** | |  |  |
|  | Fasting glucose, mean (SD) [mg/dl]/[mmol/l] | 84.3 (6.6)/4.7 (0.4) | 83.3 (8.7)/4.6 (0.5) |
|  | Fasting insulin, mean (SD) [mU/l] | 6.6 (4.1) | 5.5 (4.9) |
|  | HOMA index | 1.40 (0.97) | 1.18 (1.23) |
| ***Lipid profiles*** | |  |  |
|  | Total cholesterol, mean (SD) [mg/dl]/[mmol/l] | 167.1 (35.2)/4.3 (0.9) | 168.9 (28.4)/4.4 (0.7) |
|  | LDL cholesterol, mean (SD) [mg/dl]/[mmol/l] | 102.6 (25.5)/2.7 (0.7) | 101.3 (22.7)/2.6 (0.6) |
|  | HDL cholesterol, mean (SD) [mg/dl]/[mmol/l] | 60.3 (11.3)/1.6 (0.3) | 62.7 (13.5)/1.6 (0.3) |
|  | Triglycerides, mean (SD) [mg/dl]/[mmol/l] | 74.3 (33.1)/0.8 (0.4) | 58.7 (29.9)/0.7 (0.3) |
| ***Adipocytokines*** | |  |  |
|  | Adiponectin, mean (SD) [µg/l] | 13124.8 (3959.0) | 12876.6 (5019.9) |
|  | Leptin, mean (SD) [µg/l] | 0.77 (1.14) | 1.32 (2.11) |

Abbreviations: HDL, high-density lipoprotein; HOMA, homeostasis model assessment (index calculated as fasting insulin [mU/l] times fasting glucose [mg/dl], divided by 405); IMT, intima-media thickness; LDL, low-density lipoprotein.
