# Supplementary material for: Former Very Preterm Infants Show an Unfavorable Cardiovascular Risk Profile at a Preschool Age
Source: PLoS One. 2016 Dec 13;11(12):e0168162. doi: 10.1371/journal.pone.0168162 (PMC5154574; doi:10.1371/journal.pone.0168162)
Supplement: S2 Table — (DOCX) [file pone.0168162.s002.docx]

**S2 Table. Effect of postnatal administration of corticosteroids on cardiovascular risk indicators**

| **Variable** | | **No use of postnatal corticosteroids**  **(n=61-73)** | **Use of postnatal corticosteroids**  **(n=9-13)** |
| --- | --- | --- | --- |
| ***Blood pressure readings*** | |  |  |
|  | Systolic, mean (SD) [mmHg] | 102 (7) | 103 (7) |
|  | Mean, mean (SD) [mmHg] | 70 (7) | 70 (5) |
|  | Diastolic, mean (SD) [mmHg] | 56 (7) | 57 (9) |
| ***Aortic IMT*** | |  |  |
|  | IMT, mean (SD) [mm] | 0.460 (0.052) | 0.465 (0.078) |
| ***Glucose homeostasis*** | |  |  |
|  | Fasting glucose, mean (SD) [mg/dl]/[mmol/l] | 83.6 (8.8)/4.6 (0.5) | 80.1 (7.1)/4.4 (0.4) |
|  | Fasting insulin, mean (SD) [mU/l] | 5.6 (5.0) | 4.8 (3.6) |
|  | HOMA index | 1.21 (1.25) | 0.99 (0.79) |
| ***Lipid profiles*** | |  |  |
|  | Total cholesterol, mean (SD) [mg/dl]/[mmol/l] | 171.89 (28.9)/4.4 (0.7) | 156.2 (24.4)/4.0 (0.6) |
|  | LDL cholesterol, mean (SD) [mg/dl]/[mmol/l] | 103.5 (22.7)/2.7 (0.6) | 89.5 (17.5)/2.3 (0.5) |
|  | HDL cholesterol, mean (SD) [mg/dl]/[mmol/l] | 63.5 (14.4)/1.6 (0.4) | 62.6 (11.3)/1.6 (0.3) |
|  | Triglycerides, mean (SD) [mg/dl]/[mmol/l] | 61.8 (31.6)/0.7 (0.4) | 47.0 (14.6)/0.5 (0.2) |
| ***Adipocytokines*** | |  |  |
|  | Adiponectin, mean (SD) [µg/l] | 12635.0 (5037.6) | 13430.3 (3977.7) |
|  | Leptin, mean (SD) [µg/l] | 1.30 (2.00) | 1.03 (2.14) |

Abbreviations: HDL, high-density lipoprotein; HOMA, homeostasis model assessment (index calculated as fasting insulin [mU/l] times fasting glucose [mg/dl], divided by 405); IMT, intima-media thickness; LDL, low-density lipoprotein.
